# MSC-Derived Apoptotic Vesicles Restore Bone Marrow Niche Homeostasis in Postmenopausal Osteoporosis by miRNA-Mediated Suppression of MAPK and NF-κB Signaling Nodes

**DOI:** 10.3390/ph19050777

**Published:** 2026-05-15

**Authors:** Zhiwen Tu, Haolin Wu, Youxi Jiang, Xinxin Li, Zhiqing Huang, Songtao Shi, Ruibao Ren

**Affiliations:** 1 International Center for Aging and Cancer, Hainan Medical University, Haikou 571199, China; 2 Hospital of Stomatology, Guanghua School of Stomatology, Sun Yat-Sen University, Guangzhou 510055, China; 3 Department of Temporomandibular Joint, School and Hospital of Stomatology, Guangdong Engineering Research Center of Oral Restoration and Reconstruction & Guangzhou Key Laboratory of Basic and Applied Research of Oral Regenerative Medicine, Guangzhou Medical University, Guangzhou 510180, China

**Keywords:** apoptotic vesicles, exosomes, osteoporosis, senescence-associated secretory phenotype (SASP), microRNA, bone marrow mesenchymal stem cells

## Abstract

**Background**: Postmenopausal osteoporosis is associated with cellular senescence and the accumulation of the senescence-associated secretory phenotype (SASP). While mesenchymal stem cell (MSC)-derived exosomes show tissue repair potential, the efficacy and mechanisms of MSC-derived apoptotic vesicles (apoVs) remain unclear. This study compared MSC-apoVs and exosomes in postmenopausal osteoporosis and investigated the underlying epigenetic mechanisms. **Methods**: Therapeutic efficacy was evaluated in an ovariectomized (OVX) mouse model and senescent human bone marrow mesenchymal stem cells (hBMMSCs). Small RNA sequencing identified differential microRNA (miRNA) cargos between vesicle types. SASP-related cytokine expression (IL-6, TNF-α, MCP-1) and pathway activation were assessed by RT-qPCR, ELISA, and Western blot. **Results**: MSC-apoV treatment attenuated bone loss in OVX mice and reduced SASP expression in senescent hBMMSCs to a greater extent than exosomes. Small RNA sequencing revealed that apoVs were enriched with a specific miRNA cluster, including hsa-let-7b-5p, hsa-miR-92a-3p, and hsa-miR-98-5p. Bioinformatic analyses identified BRAF and CRKL as downstream targets of this miRNA cluster, supported by reduced protein levels after apoV treatment. Subsequent molecular assays showed that apoV treatment inhibited the phosphorylation of both the MAPK (p38 and JNK) and NF-κB (p65) signaling pathways, which correlated with reduced local inflammation in the bone marrow microenvironment and preserved osteogenic differentiation capacity. **Conclusions**: MSC-apoVs attenuate postmenopausal osteoporosis more effectively than exosomes. This enhanced efficacy is associated with the delivery of an enriched miRNA cluster that inhibits MAPK and NF-κB signaling, together with suppression of BRAF and CRKL protein expression. ApoVs may represent a cell-free therapeutic strategy for age-related bone loss.

## 1. Introduction

The increasing aging population has led to a rise in age-related skeletal disorders, including postmenopausal osteoporosis (PMOP). PMOP is a systemic skeletal disease characterized by low bone mass and deteriorated microarchitecture, contributing to approximately 17.3 million disability-adjusted life years (DALYs) worldwide according to the Global Burden of Disease (GBD) 2021 study [1,2]. Systemic ‘inflammaging’—a chronic, low-grade inflammatory state—contributes to PMOP pathogenesis [3]. This condition is associated with the accumulation of senescent cells in the bone marrow microenvironment, which acquire a Senescence-Associated Secretory Phenotype (SASP) and secrete proinflammatory cytokines including IL-6, TNF-α, and MCP-1 [4]. These factors disrupt bone homeostasis by activating inflammatory cascades including NF-κB and MAPK pathways [5].

Mesenchymal stem cell (MSC)-derived exosomes have been investigated as a cell-free therapy for bone regeneration [6]. These small extracellular vesicles (30–150 nm) facilitate horizontal transfer of bioactive molecules and have shown potential in preclinical models [7]. However, several challenges limit their clinical translation. Exosomes are products of basal cellular communication, and their molecular cargo may be insufficient to counteract the complex SASP networks in PMOP [8]. Furthermore, exosome isolation yields are typically low, limiting large-scale clinical application [9]. Alternative vesicle populations with higher yields and immunomodulatory profiles may offer advantages for treating the osteoporotic bone marrow microenvironment.

In contrast to the constitutive secretion of exosomes, apoptotic vesicles (apoVs) are generated during programmed cell death and represent a mechanism for maintaining tissue homeostasis [10]. We have previously proposed that apoptosis serves a physiological function beyond cell clearance; apoptotic vesicles compartmentalize immunomodulatory and tissue-repairing molecules that maintain systemic balance. Studies have shown that MSC-derived apoVs can regulate distant stem cell populations via systemic circulation. Specifically, MSCs can engulf apoVs via integrin αvβ3 and reuse vesicular cargos such as RNF146 and miR-328-3p to activate the Wnt/β-catenin pathway and restore bone formation [11]. Compared to exosomes, apoVs have higher production yields and a cargo profile shaped by the apoptotic process, which may contribute to systemic homeostasis [6,11].

Despite the potential of apoVs, several questions remain. First, the efficacy of MSC-derived apoVs compared to exosomes in treating PMOP has not been evaluated in a direct comparison. Second, while apoVs carry functional miRNAs, the mechanisms by which these miRNAs regulate the SASP-driven inflammatory networks (MAPK and NF-κB pathways) have not been identified [12]. Given the involvement of multiple signaling cascades in PMOP, identifying miRNAs within apoVs that target these pathways may aid in developing cell-free interventions [13].

Therefore, this study compared the therapeutic effects of MSC-derived apoVs and exosomes in an ovariectomized (OVX) murine model and senescent hBMMSCs. We used an optimized gradient centrifugation procedure to isolate vesicle populations and evaluated their capacity to restore bone mineral density and suppress systemic inflammation. Furthermore, we used small RNA sequencing and bioinformatic analysis to identify miRNA cargos in apoVs. We then validated by Western blot whether these miRNAs regulate the MAPK and NF-κB pathways, providing a mechanistic basis for using MSC-apoVs in osteoporosis treatment.

## 2. Result

### 2.1. MSC-Derived apoVs Exhibit Distinct Physicochemical Properties and Better Yield Compared to Exosomes

To obtain specific extracellular vesicle (EV) subpopulations, human umbilical cord mesenchymal stem cells (hUCMSCs) were subjected to culture conditions to induce the release of exosomes or apoptotic vesicles (apoVs).

**Figure 1:**
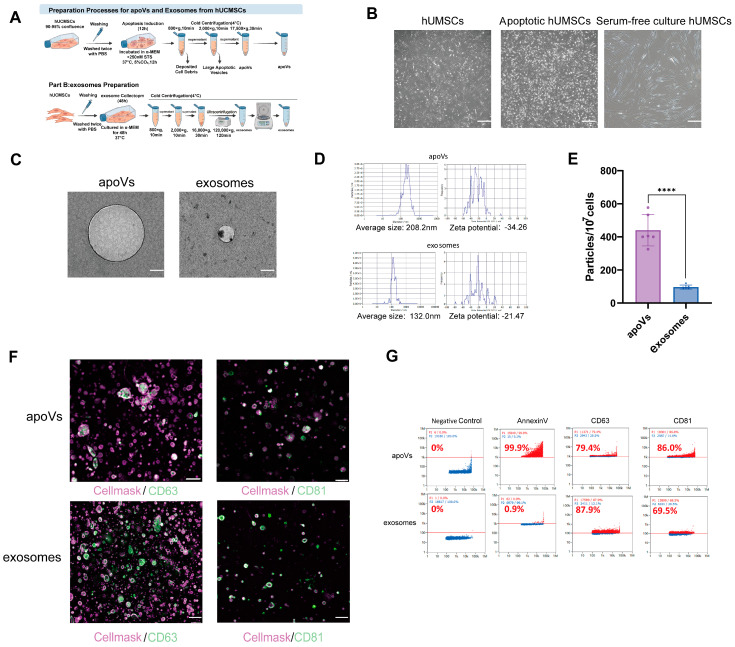
Characterization of MSC-apoVs and exosomes. (**A**) Schematic diagram illustrating the extraction and isolation workflow for MSC-apoVs and exosomes. (**B**) Representative images of MSCs under normal culture conditions, undergoing apoptosis, and following serum-free medium change. Scale bar: 100 μm for MSCs and apoptotic MSCs, 40 μm for serum-free cultured MSCs. (**C**) Transmission electron microscopy (TEM) images showing the morphology of MSC-apoVs and exosomes. Scale bar: 100 nm. (**D**) Nanoparticle tracking analysis (NTA) showing the mean size distribution and zeta potential of MSC-apoVs and exosomes. (**E**) Quantitative comparison of the production yield between the two types of extracellular vesicles (particles/10^7^ cells), *n* = 6 per group. (**F**) Immunofluorescence staining of MSC-apoV markers (CD9, CD63, and CD81) (100× magnification; Scale bar: 2 µm). (**G**) Nano flow cytometry analysis detecting the expression levels of CD63, CD81, and Annexin V in MSC-apoVs and exosomes (**** *p* < 0.0001).

We first monitored the morphological changes in the parental cells during the induction phase. UCMSCs cultured in serum-free medium for 48 h (the condition for exosome collection) maintained their typical spindle-shaped, fibroblast-like morphology with intact cell membranes, showing no signs of cytotoxicity, though the cells overall became more elongated. In contrast, upon treatment with the apoptosis-inducing reagent 250 nM staurosporine, the cells underwent morphological alterations characteristic of apoptosis. These included cell shrinkage, nuclear condensation, and most notably, extensive plasma membrane blebbing, which precedes the release of apoVs (Figure 1B). Following the collection and purification of supernatants from these cellular states, we characterized the isolated vesicles. Transmission electron microscopy (TEM) confirmed that the vesicles derived from the apoptotic cells (apoVs) were heterogeneous and membrane-bound, with generally larger dimensions. This was morphologically different from the exosomes isolated from the serum-free culture cells, which displayed a uniform, cup-shaped structure (Figure 1C). Nanoparticle tracking analysis (NTA) further quantified the physical differences. Exosomes displayed a narrow size distribution peaking between 100 and 150 nm, with a mean diameter of 132.0 nm and a Zeta potential of −21.47 mV. In contrast, apoVs exhibited a broader size distribution profile, characterized by a mean diameter of 208.2 nm and a Zeta potential of −34.26 mV, consistent with the inherent heterogeneity of apoVs (Figure 1D). Regarding production efficiency, apoVs showed a quantitative advantage. Starting from the same baseline of 1 × 10^7^ cells, the yield of exosomes was approximately 9.6 × 10^9^ particles. In comparison, the apoV yield reached 4.4 × 10^10^ particles, representing a 4.6-fold increase in particle number (Figure 1E). Given the significantly larger average volume of apoVs compared to exosomes, this quantitative increase suggests a substantially expanded total capacity for bioactive cargo delivery, underscoring their potential for applications in translational medicine. Finally, we verified the EVs identity using immunofluorescence (IF) and Nano-flow Cytometry (NanoFCM). IF staining showed CD63 and CD81 signals on both vesicle types (Figure 1F). To validate these findings at the single-particle level, we performed NanoFCM analysis. The results confirmed that apoVs, like exosomes, were positive for CD63 and CD81, indicating that they retain the canonical EV immunophenotype despite their distinct biogenic origins. Crucially, as products of apoptosis, apoVs showed distinct positivity for Annexin V, whereas exosomes were negative, confirming Annexin V as one of the specific discriminators between the two vesicle populations (Figure 1G).

### 2.2. Cellular Uptake and Rejuvenation Effects of MSC-apoVs and Exosomes on Aged hBMMSCs

**Figure 2:**
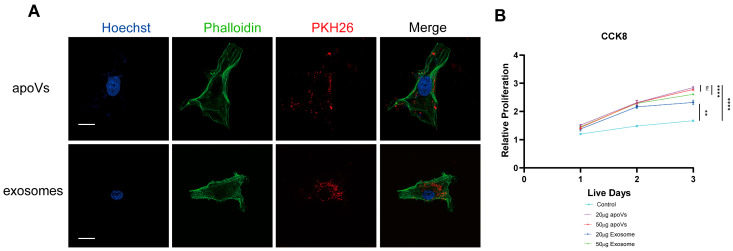
Internalization of MSC-derived apoptotic vesicles (MSC-apoVs) and their dose-dependent effects on the viability of aged hBMMSCs. (**A**) Representative Structured Illumination Microscopy (SIM) image demonstrating the cellular uptake of MSC-apoVs by aged hBMMSCs. MSC-apoVs were labeled with the lipophilic membrane dye PKH26 (red) and co-cultured with aged hBMMSCs for 24 h. The high-resolution image reveals the punctate intracellular distribution of the vesicles within the recipient cells. Scale bar: 10 µm. Original magnification: 100×. (**B**) Optimization of MSC-apoVs dosage for aged hBMMSC treatment assessed by CCK-8 assay. Data are presented as mean ± SD from three independent experiments (*n* = 3). Statistical significance was determined using one-way ANOVA (** *p* < 0.01, **** *p* < 0.0001 and ns: not significant).

After verifying the stem cell properties of aged hBMMSCs (Figure S1), we investigated the cellular uptake of MSC-apoVs and exosomes by aged hBMMSCs. MSC-apoVs and exosomes were labeled with the lipophilic membrane dye PKH26 and co-cultured with the recipient cells for 24 h. High-resolution Structured Illumination Microscopy (SIM) revealed a punctate distribution of red fluorescence within the cytoplasm (Figure 2A). This observation confirms that aged hBMMSCs successfully internalize these EVs. Optimization of the working concentration was performed by assessing the viability of aged hBMMSCs at 24, 48, and 72 h post-treatment using the CCK-8 assay (Figure 2B). Evaluation of two dosages, 20 µg/mL and 50 µg/mL, revealed that the 20 µg/mL apoV treatment elicited the most potent proliferative response. Quantitative analysis confirmed an increase in cell viability in the 20 µg/mL apoV group compared to the control (*p* < 0.0001). While the 20 µg/mL exosome treatment also enhanced cell growth relative to the control (*p* < 0.01), the therapeutic efficacy was less than apoV treatment (*p* < 0.0001). Comparison between the two apoV dosages indicated no statistical difference (ns); nevertheless, the 20 µg/mL group consistently displayed a better mean trend in efficacy. Consequently, 20 µg/mL was established as the optimal dosage for all subsequent in vitro functional assays.

**Figure 3:**
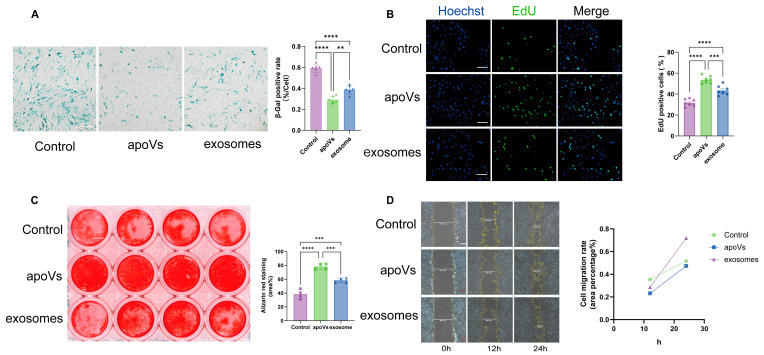
Therapeutic efficacy of apoVs and exosomes on senescent hBMMSCs. (**A**) Semi-quantitative analysis of senescence using SA-β-Gal staining, *n* = 6 per group, scale bar: 100 µm; (**B**) Proliferation detection in senescent hBMMSCs following MSC-apoV or exosome treatment using EdU assay, *n* = 7 per group. Scale bar: 100 μm; (**C**) Osteogenesis of aging hBMMSCs cocultured with MSC-apoVs or exosomes as assessed by Alizarin red staining, *n* = 4 per group, scale bar: 80 mm; (**D**) Scratch-wound assay and analysis showing hBMMSCs’ migration in different groups, scale bar: 200 μm. Error bars are mean ± SD. Data were analyzed by one-way ANOVA with Bonferroni test for comparison of multiple groups. ** *p* < 0.01, *** *p* < 0.001 and **** *p* < 0.0001.

The therapeutic efficacy of MSC-apoVs and exosomes on functional restoration was evaluated across four key dimensions: senescence, proliferation, osteogenesis, and migration. SA-β-Gal staining determined the level of cellular senescence (Figure 3A). The control group exhibited a severe senescent phenotype with a mean positive rate of 59.2%. Both vesicle treatments significantly alleviated senescence compared to the control (*p* < 0.0001). Notably, apoVs demonstrated better anti-aging efficacy, reducing the positive rate to 29.1% compared to 38.8% in the exosome group (*p* < 0.01). The EdU assay assessed cell proliferation capabilities (Figure 3B). Results were consistent with the senescence data. Both treatments significantly enhanced proliferative capacity relative to the control (*p* < 0.0001). The MSC-apoV group exhibited a more potent effect, with a significantly higher percentage of proliferating cells compared to the exosome group (*p* < 0.001), indicating a stronger rejuvenation capability. Alizarin red staining visualized calcium deposition after 24 days of osteogenic induction (Figure 3C). While the control group displayed minimal mineralization (average 38.3% stained area), the MSC-apoV treatment induced the most osteogenesis (average 78.3% area). This response was significantly higher than that of the exosome group (average 58.4% area, *p* < 0.001), although both treatments outperformed the control (*p* < 0.0001). Finally, we analyzed cell migration behavior using a scratch wound-healing assay (Figure 3D). The results revealed a distinct pattern compared to the previous assays. The exosome treatment significantly accelerated the relative migration rate compared to the control. Conversely, the apoV group exhibited a reduced migration capacity, with a rate lower than the control group. This divergence suggests that while apoVs are better in promoting proliferation and differentiation, exosomes are more effective in enhancing cell motility. This functional divergence implies distinct mechanisms of action between the two vesicle populations. These findings indicate that both types of EVs exert positive effects on hBMMSCs. MSC-apoVs demonstrate greater efficacy in promoting proliferation and differentiation compared to exosomes. They also exhibit stronger anti-aging effects. In contrast, exosomes show higher potential in enhancing cell migration.

### 2.3. Comparative Analysis of the Therapeutic Efficacy of apoVs and Exosomes in the OVX Mice

**Figure 4:**
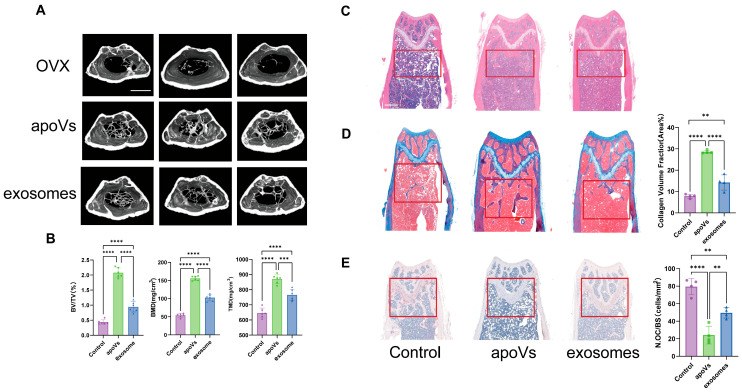
Comparative Analysis of the therapeutic efficacy of apoVs and exosomes in OVX mice. (**A**,**B**) MicroCT images and analysis of the distal femurs in OVX mice (the control group is OVX mice without any treatment), *n* = 6 per group, scale bar: 1 mm; Representative (**C**) H&E, (**D**) Masson’s trichrome, and (**E**) TRAP staining and analysis of distal femoral sections from OVX mice (4× objective), *n* = 4 per group. Scale bars: 250 μm (H&E and Masson’s trichrome staining); 125 μm (TRAP staining). Error bars are mean ± SD. Data were analyzed by one-way ANOVA with Bonferroni test for comparison of multiple groups. ** *p* < 0.01, *** *p* < 0.001, and **** *p* < 0.0001. The red boxes highlight the representative regions for visual comparison.

MicroCT imaging assessed the bone microarchitecture of distal femurs (Figure 4A). The OVX group displayed severe bone loss compared to the Control group. Quantitative analysis confirmed this deterioration (Figure 4B). The bone volume fraction (BV/TV) in the OVX group dropped to 0.43%. Administration of apoVs or exosomes rescued this phenotype, but apoVs demonstrated better osteoprotective effects compared to exosomes. The apoV group achieved a BV/TV of 2.07%, whereas the exosome group reached 0.94% (*p* < 0.0001). Similar trends appeared in bone mineral density (BMD) and tissue mineral density (TMD).

We evaluated the therapeutic efficacy of apoVs and exosomes in vivo using the ovariectomized (OVX) mouse model. Histological analysis provided further confirmation of bone repair. H&E staining visualized the trabecular microstructure (Figure 4C). The OVX group displayed sparse and fragmented trabeculae. Conversely, the apoV treatment preserved trabecular integrity. This group exhibited increased trabecular number and density. The exosome group maintained a relatively intact architecture compared to the control. However, the trabecular quantity was lower than that of the apoV group. Masson’s trichrome staining assessed new bone formation and collagen deposition (Figure 4D). Both apoV and exosome treatments significantly increased collagen tissue formation compared to the control. The exosome group showed a significant improvement (*p* < 0.01). However, the apoV group demonstrated the most osteogenic effect. It displayed a better new bone formation area of 28.6%. This value was significantly higher than the 14.3% in the exosomes group (*p* < 0.0001) and the 7.8% in the control group (*p* < 0.0001). TRAP staining evaluated osteoclast activity and bone resorption status (Figure 4E). We quantified the Number of Osteoclasts per Bone Surface (N.Oc/BS). The OVX group exhibited elevated osteoclastic bone resorption. The mean N.Oc/BS value reached 79.4 cells/mm^2^. Both therapeutic interventions significantly suppressed osteoclast formation compared to the control. However, the apoV group demonstrated the most potent inhibition. It showed the lowest N.Oc/BS value of 24.1 cells/mm^2^. This was significantly lower than the 49.6 cells/mm^2^ observed in the exosome group (*p* < 0.01). These findings confirm the therapeutic potential of both vesicle types in the OVX model. ApoVs demonstrate greater osteoprotective efficacy compared to exosomes. Specifically, apoVs more effectively restore trabecular microarchitecture and promote bone formation. Furthermore, apoVs exhibit a stronger capacity to inhibit osteoclast-mediated bone resorption than exosomes.

### 2.4. Distinct miRNA Signature of MSC-apoVs Compared to Exosomes

Sequencing and differential analysis small RNA sequencing profiled the miRNA landscapes of MSC-apoVs and exosomes.

**Figure 5:**
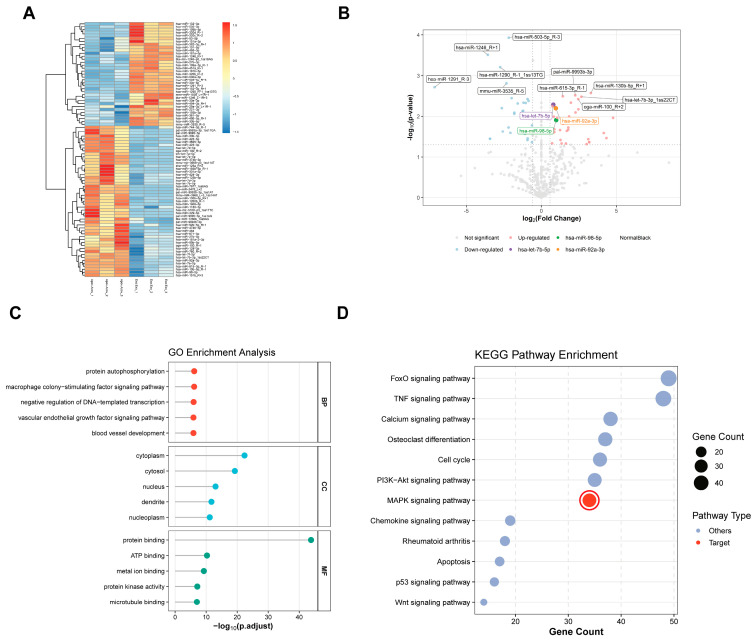
Differential expression profile and functional enrichment analysis of miRNAs in apoVs and exosomes. (**A**) Hierarchical clustering heatmap of the differentially expressed miRNAs (DEMs). Columns represent individual samples, and rows represent miRNAs. The color scale indicates relative expression levels (red: high; blue/green: low). The clustering dendrograms show distinct separation between apoVs and exosomes. (**B**) Volcano plot displaying the statistical significance versus fold change of the miRNAs. Red and blue dots indicate significantly upregulated and downregulated miRNAs, respectively (Adjusted *p* < 0.05, |log_2_ FC| > 0.58), while gray dots represent non-significant miRNAs. (**C**) Lollipop chart of the top 5 significantly enriched Gene Ontology (GO) terms for the target genes of DEMs, categorized into Biological Process (BP), Cellular Component (CC), and Molecular Function (MF). (**D**) Bubble chart showing the top 20 enriched KEGG signaling pathways. The size of each bubble corresponds to the number of enriched genes, and the color gradient indicates the significance of enrichment (*p*-value).

To visualize the distinct regulatory patterns between groups, we performed hierarchical clustering analysis on the differentially expressed miRNAs. To ensure accurate comparison across biological replicates, the normalized expression values were standardized using Z-scores. The resulting heatmap (Figure 5A) visualizes the relative expression intensity, where the color gradient transitions from dark blue (low expression) to red (high expression). This analysis revealed a clear segregation between samples. MSC-apoVs and exosomes clustered into independent branches, indicating that apoVs possess a unique miRNA expression profile distinct from that of exosomes. The heatmap identified a specific cluster of miRNAs that were highly enriched (indicated by red) in the MSC-apoV group but depleted (dark blue) in the exosome group. This cluster includes prominent pro-osteogenic miRNAs such as hsa-let-7b-5p, hsa-miR-98-5p, and hsa-miR-92a-3p. Conversely, another cluster containing hsa-miR-30a-5p and hsa-miR-181a-5p showed the opposite pattern, being highly abundant in exosomes but barely detectable in apoVs. This distinct “red-vs-blue” signature suggests a selective packaging mechanism unique to the apoptotic process. To systematically identify the specific miRNA signature of MSC-apoVs, we performed differential expression analysis with statistical thresholds set at adjusted *p* < 0.05 and |log_2_FoldChange| > 0.58. The volcano plot (Figure 5B) visualizes the distribution of the miRNA profiles. The analysis revealed a dynamic regulation pattern: 33 miRNAs were significantly upregulated (depicted in soft red) and 28 miRNAs were downregulated (depicted in soft blue) in MSC-apoVs compared to exosomes. Non-significant miRNAs were shown in gray. We specifically scrutinized three candidate miRNAs associated with the MAPK pathway. As highlighted in the plot, hsa-let-7b-5p (purple), hsa-miR-98-5p (green), and hsa-miR-92a-3p (orange) were distinct from the general cluster. Among them, hsa-let-7b-5p exhibited the most prominent enrichment pattern, combining high statistical significance with a substantial fold change.

To further categorize the functions of the apoV-specific miRNA signature, we performed GO annotation analysis. The lollipop plot (Figure 5C) displays the top 5 most significantly enriched terms within three categories: Biological Process (BP), Cellular Component (CC), and Molecular Function (MF). In the BP category (marked in red), the upregulated miRNAs were primarily involved in signaling regulation and vascular maintenance. Key enriched terms included protein autophosphorylation, macrophage colony-stimulating factor signaling pathway, and blood vessel development. This aligns with the potential of MSC-apoVs to modulate kinase activity and angiogenesis. In the CC category (marked in blue), the targets were localized to cytoplasm and cytosol. In the MF category (marked in green), the identified miRNAs showed molecular activities related to protein binding and ATP binding. These GO signatures indicate that MSC-apoVs are functionally distinct from exosomes, specifically in regulating phosphorylation-dependent developmental processes. To identify the specific signaling cascades driving these biological processes, we conducted KEGG pathway analysis. The bubble plot (Figure 5D) visualizes the enriched signaling pathways. Top-ranked categories, including PI3K-Akt, FoxO, Cell cycle, and Calcium signaling, represent broad regulatory modules governing cell proliferation and survival. This aligns with the better pro-proliferative effect of apoVs observed in our CCK-8 assays. Beyond these general growth signals, we identified the MAPK signaling pathway as a specific regulator of osteogenic differentiation. Although MAPK ranked behind the broad survival pathways in gene count, it showed statistical significance (*p* < 0.05). Furthermore, the MAPK cascade mechanistically corresponds to the “protein autophosphorylation” signature identified in our GO analysis. Therefore, we prioritized the MAPK axis to delineate the specific osteoinductive mechanism of MSC-apoVs.

### 2.5. MSC-apoV-Derived miRNAs Target a Dual-Channel Network to Inhibit SASP

**Figure 6:**
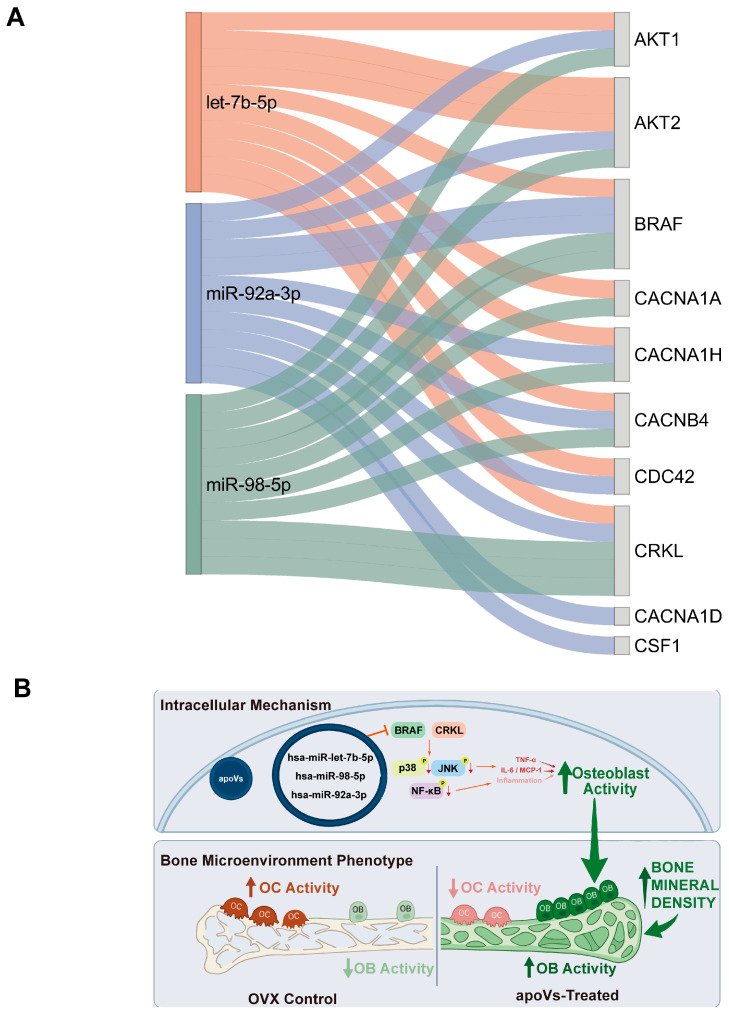
Elucidation of the specific miRNA-MAPK/NF-κB regulatory axis and the proposed working model. (**A**) Sankey diagram illustrating the targeted regulatory network connecting the three candidate miRNAs (hsa-let-7b-5p, hsa-miR-98-5p, and hsa-miR-92a-3p) to their downstream targets within the MAPK signaling cascade. The flow width corresponds to the number of predicted interactions, explicitly linking miRNAs (left) to key MAPK pathway components, thereby highlighting the multi-target potential of apoV cargo. (**B**) Schematic representation of the proposed “dual-pathway inhibition” mechanism by which MSC-apoVs ameliorate cellular senescence. Upon internalization by senescent hBMMSCs, MSC-apoVs deliver a core miRNA cluster (let-7b-5p, miR-98-5p, and miR-92a-3p) that synergistically targets the signaling hub genes BRAF and CRKL. This targeted silencing concurrently dampens the phosphorylation and pathological activation of both the MAPK (p38/JNK) and NF-κB (p65) signaling cascades. Consequently, the intracellular expression of senescence markers (p16, p21) is downregulated, and the hypersecretion of core Senescence Associated Secretory Phenotype (SASP) factors (IL-6, TNF-α, and MCP-1) is profoundly mitigated, ultimately orchestrating the rejuvenation of the osteogenic microenvironment.

Our KEGG and GO analyses prioritized the MAPK signaling axis and a “protein autophosphorylation” signature. To identify the upstream epigenetic drivers of these pathway alterations, we investigated the miRNA cargo of MSC-apoVs. Small RNA sequencing revealed three specific miRNAs—hsa-let-7b-5p, hsa-miR-92a-3p, and hsa-miR-98-5p—with exceptionally high abundance, exhibiting normalized expression levels up to over 10,000 TPM and ranking strictly within the top 8% of all detected miRNAs. Target gene prediction for this miRNA cluster was performed using TargetScan and miRanda. Strict filtering criteria (TargetScan context score percentile ≥ 50; miRanda maximum free energy < −10 kcal/mol) were applied to generate a high-confidence intersection. The resulting miRNA-mRNA regulatory Sankey diagram (Figure 6A) revealed a strong convergent targeting pattern. The kinase BRAF and the adaptor protein CRKL emerged as core candidate nodes collectively targeted by all three enriched miRNAs.

These in silico findings prompt a theoretical working model for a “parallel dual-channel inhibitory mechanism” conferred by MSC-apoVs in senescent cells (Figure 6B). Upon cellular internalization, the released miRNA cluster is proposed to bind and suppress the translation of the BRAF/CRKL complex. This upstream blockade would deprive downstream signaling hubs of their primary activation cues, thereby attenuating two interconnected inflammatory axes. Within this framework, BRAF suppression dampens the downstream phosphorylation of the MAPK cascade (comprising stress kinases p38 and JNK). In parallel, CRKL silencing impedes the NF-κB cascade by inhibiting the IKK complex, limiting IκB degradation and subsequent nuclear translocation of the active p65 subunit. The hyperactivation of nuclear p38/JNK and p65 directly drives the transcription of the Senescence-Associated Secretory Phenotype (SASP). By disrupting these parallel channels, this targeted molecular reprogramming theoretically downregulates key pro-inflammatory cytokines, including TNF-α, IL-6, and MCP-1. This model offers a conceptual rationale for the remodeling of the osteoporotic microenvironment by MSC-apoVs. To empirically substantiate this signal-transduction logic, we next investigated the downstream molecular alterations in vitro and in vivo.

### 2.6. MSC-apoVs Mitigate the Senescence-Associated Secretory Phenotype via Targeted Dual-Channel Signal Blockade

**Figure 7:**
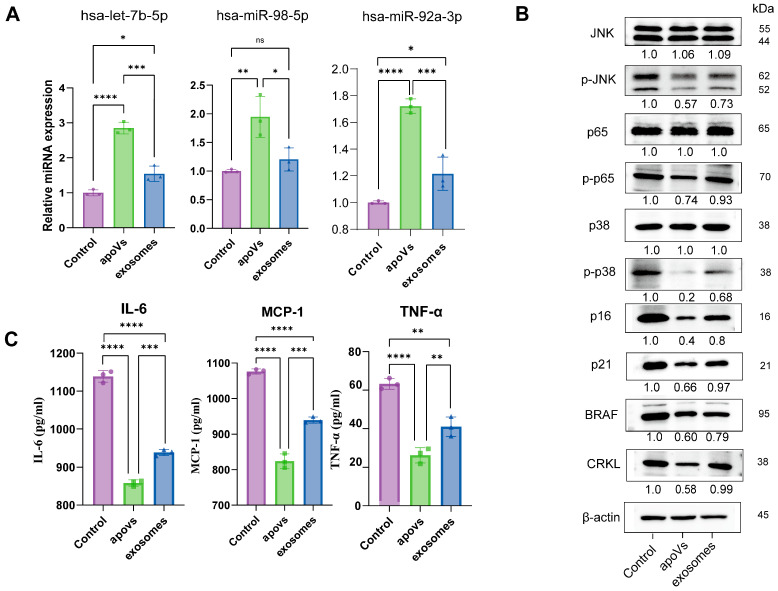
Validation of apoVs-mediated miRNA delivery and suppression of the SASP in senescent hBMMSCs. (**A**) qPCR analysis quantifying the relative expression levels of the three candidate miRNAs (hsa-let-7b-5p, hsa-miR-98-5p, and hsa-miR-92a-3p) in senescent hBMMSCs. (**B**) Representative Western blot images and densitometric quantification of BRAF, CRKL, MAPK (p-p38, total p38, p-JNK, total JNK), NF-κB (p-p65, total p65), and senescence markers (p16, p21) in the same samples. (**C**) ELISA quantification of pro-inflammatory cytokines (IL-6, TNF-α) and chemokine (MCP-1) in culture supernatants. Data are presented as mean ± SD from three independent experiments (*n* = 3). Statistical significance was determined using one-way ANOVA (* *p* < 0.05, ** *p* < 0.01, *** *p* < 0.001, **** *p* < 0.0001, ns: not significant).

To first confirm the functional delivery of the therapeutic cargo, we quantified the intracellular abundance of the candidate miRNAs (Figure 7A). qPCR revealed a marked enrichment of hsa-let-7b-5p, hsa-miR-92a-3p, and hsa-miR-98-5p in target cells following apoV treatment. This delivery efficacy was greater than that achieved with traditional exosomes. Exosome administration induced only marginal or non-significant increases in these specific miRNAs (e.g., miR-92a-3p and miR-98-5p, *p* > 0.05 vs. Control), whereas apoVs drove a highly significant miRNA influx (e.g., let-7b-5p, *p* < 0.0001 vs. Control; *p* < 0.001 vs. exosomes).

To elucidate the mechanism underlying this SASP reversal, we profiled the downstream signal transduction network by Western blot (Figure 7B). First, we examined the expression of the predicted target proteins BRAF and CRKL. ApoV treatment significantly reduced the protein levels of both BRAF and CRKL in recipient senescent hBMMSCs. Furthermore, Western blot analysis validated the predicted parallel dual-channel blockade, revealing a deactivation of both the MAPK and NF-κB signaling axes exclusively following apoV administration. The phosphorylation levels of stress kinases p38 and JNK were attenuated, concomitant with a reduction in p-p65, while their respective total protein pools remained stable. Critically, this dual-channel blockade culminated in an alleviation of cellular senescence, evidenced by the downregulation of the classical senescence markers p16 and p21.

Consistent with these findings, we next assessed the Senescence-Associated Secretory Phenotype (SASP) by ELISA (Figure 7C). ELISA of conditioned medium demonstrated a marked reduction of key pro-inflammatory cytokines following apoV administration. The hypersecretion of IL-6, MCP-1, and TNF-α characteristic of the senescent control group was suppressed to near-basal levels (*p* < 0.0001). Although exosomes provided partial suppression of these SASP factors, this inhibitory effect remained less pronounced than that of apoVs (IL-6 and MCP-1: *p* < 0.001; TNF-α: *p* < 0.01, apoVs vs. exosomes), corroborating the enhanced therapeutic potency of the apoV-derived cargo.

Together, these results indicate that MSC-apoVs deliver a specific miRNA cluster that suppresses MAPK and NF-κB signaling, accompanied by reduced BRAF and CRKL protein expression, leading to decreased SASP and alleviation of cellular senescence.

## 3. Discussion

The increasing aging population has led to a rise in age-related skeletal disorders, including postmenopausal osteoporosis (PMOP). Postmenopausal osteoporosis (PMOP), defined by low bone mass and deteriorated microarchitecture, is a major public health challenge, contributing to approximately 17.3 million disability-adjusted life years (DALYs) worldwide according to the Global Burden of Disease (GBD) 2021 study [2,14]. The biological essence of PMOP lies in a systemic imbalance of bone homeostasis, where the synchronized coupling of osteoblastic bone formation and osteoclastic bone resorption is fundamentally disrupted. We have previously proposed that aging represents not simply passive damage accumulation, but a progressive loss of physiological integrity driven by immune microenvironment deterioration and systemic “inflammaging” [11]. In the context of estrogen deficiency, the bone marrow niche undergoes a significant transformation; bone marrow mesenchymal stem cells (BMMSCs) transition from a regenerative, multipotent state into a senescent phenotype characterized by stable cell-cycle arrest and the acquisition of the SASP [15]. This SASP-mediated secretion of pro-inflammatory cytokines, chemokines, and MMPs compromises the self-renewal and differentiation potential of neighboring progenitor cells and promotes excessive osteoclastogenesis. This creates a feed-forward loop that accelerates skeletal fragility [15].

The therapeutic efficacy of MSC-derived apoVs demonstrated in this study supports the concept that apoptosis serves a physiological function beyond simply marking the end of cell life [11]. In the human body, approximately 50 to 70 billion cells undergo apoptosis every day to maintain tissue homeostasis by eliminating damaged or unwanted cells. Our previous work identified that apoptotic vesicles compartmentalize immunomodulatory signals and molecules (including Fas, ubiquitin ligases, and specific miRNAs) that support self-renewal and differentiation of endogenous stem cells [11]. The data show that MSC-apoVs are more effective than conventional exosomes in reversing OVX-induced osteoporosis and attenuating senescence in hBMMSCs. This functional superiority can be mechanistically explained by the distinct biogenesis and cargo profiles of these two vesicle populations. While exosomes are derived from the endosomal pathway during active cellular secretion, apoVs are generated during apoptosis, allowing concentration of functional proteins and RNA species that are less abundant in exosomes [16]. Consistent with their higher buoyant density (1.118–1.228 g/mL), apoVs contain complex macromolecular assemblies such as the mechanosensitive ion channel Piezo1 and the mitochondrial regulator TCOF1. Both molecules are critical for overcoming apoptotic resistance and mitochondrial dysfunction in aging BMMSCs [17]. These findings suggest that MSC-apoVs deliver a broader range of factors that modulate the senescent bone marrow niche more effectively than the transient signals mediated by exosomes [18]. We acknowledge that the secretome of living MSCs under stress conditions can also exhibit immunomodulatory properties. However, the present study focuses on a direct head-to-head comparison between apoVs and baseline exosomes harvested from untreated MSCs under standard culture conditions. The key advantage of apoVs lies in their one-time release during apoptosis, which yields substantially higher particle numbers and enriched miRNA cargo compared to conventional exosome preparations. We also recognize that while apoVs offer a higher yield per single harvest, living MSCs can be cultured for multiple passages to continuously produce exosomes. The comparison in this study is based on a single batch production from the same number of starting cells. For large-scale industrial production, the optimal method may depend on the specific manufacturing setup, and future cost-effectiveness analyses are warranted.

A key mechanistic finding of this study is the identification of a specific miRNA cluster—let-7b-5p, miR-92a-3p, and miR-98-5p—that is enriched in apoVs. Small RNA sequencing analysis indicates that the transition from a living MSC to an apoptotic state leads to selective loading of these miRNAs, a process that distinguishes apoVs from the secretory output of healthy cells [16].

The let-7 family, particularly let-7b-5p, plays a well-established role in regulating cellular longevity by suppressing oncogenic and pro-aging genes. In the cardiovascular and musculoskeletal systems, let-7b-5p levels serve as biomarkers of cellular stress and systemic aging [19]. Similarly, miR-92a-3p and miR-98-5p have been implicated in regulating the MAP kinase pathway and modulating DNA damage responses, both of which contribute to the senescent phenotype in BMMSCs [20,21,22,23]. Our findings suggest that this miRNA cluster acts synergistically to suppress the genetic programs underlying SASP acquisition. By delivering this concentrated molecular payload, MSC-apoVs attenuate the inflammatory output of senescent hBMMSCs and restore their osteogenic differentiation capacity. This selective enrichment supports the concept that apoVs function as specialized mediators in the maintenance of stem cell health during physiological cell turnover [24,25].

We acknowledge that members of the let-7 family exhibit pleiotropic effects and can target a broad network of mRNAs [26]. A limitation of this study is the lack of direct validation of miRNA-mRNA binding (e.g., by dual-luciferase reporter assays). Therefore, BRAF and CRKL serve as putative candidate nodes in this context, and we do not exclude the likely contribution of other uncharacterized downstream effectors to the observed dual pathway inhibition.

A second major finding of this study is the elucidation of how SASP clearance restores the bone remodeling cycle through simultaneous inhibition of the MAPK and NF-κB signaling nodes. p38 MAPK is a key regulator of cellular senescence and a primary activator of SASP cytokine production, including IL-6, IL-8, and MMPs [27,28,29,30]. NF-κB signaling, particularly p65 phosphorylation, is also known to promote the expression of pro-inflammatory cytokines and to contribute to osteoclastogenesis [31,32,33,34]. Our data show that MSC-apoV treatment suppresses p-p38, p-JNK, and p-p65, reducing the inflammatory output of senescent BMMSCs. Notably, the reduction in p-p38 and p-JNK occurred without changes in total p38 and JNK protein levels, indicating that apoVs inhibit the hyperactivation of MAPK induced by the senescent microenvironment rather than abolishing basal MAPK activity. This perspective is supported by a previous study demonstrating that p38 MAPK signaling is essential for osteoblast differentiation [35]. Therefore, the selective inhibition of excessive MAPK activation by apoVs likely preserves the essential basal signaling while attenuating pathological inflammation. Similarly, apoVs reduced p-p65 levels without affecting total p65 expression, suggesting selective suppression of stress-induced NF-κB activation. Basal NF-κB activity, which is critical for cell survival and normal bone remodeling, is likely maintained [33]. This selectivity may explain why apoVs effectively reduce inflammation-driven bone resorption without causing unintended cytotoxicity. It is well established that excessive osteoclast activity is the primary driver of bone resorption and skeletal deterioration in postmenopausal osteoporosis [36]. Consistent with this, TRAP staining revealed significantly reduced osteoclast numbers in apoVs-treated mice. While our mechanistic data focus on the suppression of inflammatory pathways that drive osteoclastogenesis, the ultimate therapeutic goal is to restore bone formation by improving the osteogenic microenvironment. The reduction in SASP factors (IL-6, TNF-α, MCP-1) likely contributes to both decreased bone resorption and enhanced osteoblast function. Thus, the anti-inflammatory effects of apoVs indirectly support osteogenesis, which is consistent with the observed preservation of trabecular bone mass. This dual inhibition contributes to restoring the coupling between osteoblasts and osteoclasts, which is impaired in the postmenopausal state. By reducing SASP, apoVs diminish the paracrine signals that inhibit osteoprogenitor differentiation and stimulate bone resorption, shifting the bone marrow microenvironment from a pro-resorptive state toward a more homeostatic state [17,37,38]. This mechanism suggests that apoVs function not only as delivery systems but also as senomorphic interventions that modulate the aging skeletal niche [11].

Through bioinformatic predictions and subsequent Western blot validation, we identified BRAF and CRKL as downstream effectors of the miRNA cluster. Both proteins were significantly reduced in recipient cells after apoV treatment (Figure 7B). BRAF is a crucial kinase in the RAS-RAF-MAPK signaling cascade, and its aberrant activation is frequently linked to cellular proliferation defects and the induction of senescence-like states [39,40,41]. Similarly, CRKL is a key adaptor protein that facilitates the activation of both the RAS and JNK pathways, effectively bridging extracellular signals to the master regulators of inflammation and cell cycle arrest [39,40,41]. The observed suppression of these two proteins provides a mechanistic link between the upstream miRNA enrichment in apoVs and the experimentally validated downstream dual inhibition of MAPK and NF-κB. Although direct binding between the miRNA cluster and the 3′UTRs of BRAF/CRKL remains to be confirmed by luciferase reporter assays, the consistent reduction in their protein levels supports their functional involvement. These findings align with a framework of systemic homeostasis and physiological clearance in aging and bone metabolism that we have previously proposed [11], where apoptotic vesicles function as physiological mediators that preserve tissue integrity during cell turnover.

We recognize that the use of 10-week-old OVX mice does not perfectly simulate aged postmenopausal osteoporosis. This model is standard for initial efficacy evaluation, and our in vitro senescent hBMSC model partially addresses age-related aspects. Future studies in aged animals are needed to confirm the therapeutic potential of apoVs. Additionally, we acknowledge that replicative senescence in vitro (passages 21–24) does not fully capture the complex, multi-factorial inflammaging seen in human postmenopausal osteoporosis. Nevertheless, this model is widely used for mechanistic studies of cellular aging, and our findings provide proof of concept that apoVs can reverse senescence-associated phenotypes. Future validation in primary cells from aged donors or in aged animal models is needed.

In conclusion, this study demonstrates that MSC-derived apoptotic vesicles (apoVs) are effective in treating postmenopausal osteoporosis in a preclinical model. We show that MSC-apoVs deliver a specific miRNA cluster that suppresses SASP and inhibits MAPK and NF-κB signaling in the bone marrow microenvironment. These findings suggest that cell-free biological products may offer an alternative to traditional pharmacological inhibitors for managing the skeletal imbalance in PMOP. Future studies are needed to validate the direct targeting of BRAF and CRKL and to further elucidate the mechanisms underlying apoVs-mediated bone regeneration. Such work will be important for assessing the clinical potential of MSC-apoVs for age-related bone diseases.

## 4. Materials and Methods

### 4.1. Animal Welfare

Thirty female SPF-grade C57BL/6 mice (8 weeks old) were obtained from SPF (Beijing, China) Biotechnology Co., Ltd. and housed at the Hainan Provincial Pharmaceutical Research and Development Science Park, China. Housing included Level II conditions, a 12-h light/dark cycle, a temperature of 23–26 °C, 45–60% relative humidity, and ad libitum access to food and water. The ethics approval number was (HYPLL-2026-002). Euthanasia was performed via cervical dislocation, involving neck extension and firm pressure to fracture the cervical vertebrae and sever the spinal cord, ensuring a rapid and humane death.

### 4.2. Animal Model Treatment

Ten-week-old C57BL/6 mice were anesthetized by intraperitoneal injection of Tribromoethanol (10 mg/kg). A 1.5 cm longitudinal incision was then made along the dorsal midline using ophthalmic scissors. Subcutaneous tissue was bluntly dissected, and the fascia and lateral abdominal muscles were incised. The ovaries were excised, and hemostasis was secured with electrocautery. Tissues were repositioned, and the muscle and skin layers were sutured. After recovering from anesthesia, the mice were returned to their cages for routine care. Femur X-rays were taken 12 weeks post-surgery to verify the model.

### 4.3. Cell Culture

This study was approved by the Medical Ethics Committee of the School of Stomatology, Sun Yat-sen University (KQEC-2021-59-01). As described in our previous study [42], tissue samples were obtained from donors during full-term cesarean sections following informed consent. After rinsing and removal of blood vessels, the umbilical cord tissue was minced into small fragments and digested with Collagenase I (2 mg/mL; Worthington Biochemical, Lakewood, NJ, USA) and Collagenase II (4 mg/mL; Roche Diagnostics, Mannheim, Germany) at 37 °C for 1 h. The digest was then filtered through a 70-µm cell strainer (BD Biosciences, San Jose, CA, USA) to obtain a single-cell suspension. Nucleated cells were seeded onto 100-mm culture dishes (BIOFIL, Guangzhou, China) and maintained in α-MEM (Gibco, Carlsbad, CA, USA) supplemented with 15% fetal bovine serum (FBS; NEWZERUM, Christchurch, New Zealand), 2 mM L-glutamine (Gibco, Grand Island, NY, USA), and 1% penicillin/streptomycin (Invitrogen, Carlsbad, CA, USA) at 37 °C in a humidified atmosphere with 5% CO_2_. The culture medium was replaced every 3 days. hUCMSCs from passages 5 to 10 were utilized for subsequent experiments.

Human bone marrow-derived mesenchymal stem cells (hBMMSCs) were obtained from ScienCell Research Laboratories (Cat. #7500; Carlsbad, CA, USA). These cells were cultured under conditions identical to those described above for hUCMSCs. hBMMSCs at passages 21 to 24 were utilized for this study.

### 4.4. Characterization of hBMMSCs

The immunophenotypic characterization of hBMMSCs was performed via flow cytometry using a Cytek Aurora system (Cytek Biosciences, Fremont, CA, USA). Briefly, hBMMSCs were harvested and resuspended in Stain Buffer (BD Pharmingen, San Diego, CA, USA) at a concentration of 5 × 10^5^ cells/mL. The cell suspension was incubated at 4 °C for 30 min in the dark with the following antibodies (all from BioLegend, San Diego, CA, USA; 1:100 dilution): FITC-conjugated anti-human CD73, CD90, and CD105; PE-conjugated anti-human CD34; APC-conjugated anti-human CD45; and 7-AAD for viability assessment. Data acquisition and analysis were conducted using SpectroFlo^®^ software (version 3.3.0, Cytek Biosciences, Fremont, CA, USA).

### 4.5. Induction of hUCMSC Apoptosis and Isolation of apoVs

To induce apoptosis, hUCMSCs at 90–95% confluence were washed twice with PBS (Gibco, USA) and incubated in α-MEM containing 250 nM staurosporine (STS; Enzo Life Sciences, Farmingdale, NY, USA) for 12 h at 37 °C in 5% CO_2_. According to our previous protocol [43], apoVs were isolated from the apoptotic MSC culture medium by sequential centrifugation at 4 °C (800× *g* for 10 min, 2000× *g* for 10 min, and 17,500× *g* for 30 min). Finally, the apoVs were purified by washing once with 0.22-μm filtered PBS.

### 4.6. Isolation of Exosomes

As in our previous report [16], hUCMSCs were washed twice with PBS and cultured in α-MEM for 48 h at 37 °C. exosomes were then isolated from the culture supernatants by sequential centrifugation at 4 °C: 800× *g* for 10 min, 2000× *g* for 10 min, 16,000× *g* for 30 min, and finally 120,000× *g* for 120 min, in strict accordance with the MISEV2023 guidelines [44].

### 4.7. Characterization of apoVs and Exosomes

For nano-flow cytometric analysis, aliquots of isolated apoVs and exosomes were transferred to microcentrifuge tubes and resuspended in Annexin V Binding Buffer (BD Biosciences, USA). The samples were incubated in the dark at 4 °C for 30 min with the following FITC-conjugated reagents (all from BioLegend, USA; 1:100 dilution): anti-human CD63, anti-human CD81, and Annexin V. Analysis was performed using a Flow NanoAnalyzer (NanoFCM Inc., Xiamen, China) according to the manufacturer’s protocol, maintaining an optimal particle count of 4000–8000 events.

For Nanoparticle Tracking Analysis (NTA), apoVs and exosomes were diluted to appropriate concentrations in 0.22-μm filtered PBS. Measurements were recorded at 11 distinct positions using a ZetaView PMX120 system (Particle Metrix, Holly Springs, NC, USA) according to the manufacturer’s instructions. The particle number, diameter, and Zeta potential of both apoVs and exosomes were analyzed using ZetaView software (version 8.05.16 SP7).

The ultrastructural morphology of apoVs and exosomes was examined using a transmission electron microscope (TEM). Briefly, a 20 μL aliquot of the suspension was deposited onto a carbon-coated copper grid and incubated for 5 min. Excess liquid was removed by blotting with filter paper. The grid was then negatively stained with 2% phosphotungstic acid (Servicebio, Wuhan, China) for 1–2 min. After blotting the excess stain and air-drying at room temperature, the specimens were imaged using a transmission electron microscope (Hitachi, Tokyo, Japan).

For super-resolution imaging, apoVs and exosomes were stained with FITC-conjugated anti-human CD63 and CD81 antibodies (1:100 dilution) at 4 °C for 20 min, followed by membrane labeling with CellMask™ (1:1000 dilution; Invitrogen, USA) at room temperature for 10 min. To remove unbound dyes, samples were centrifuged at 17,000× *g* for 10 min. The labeled particles were resuspended and visualized using a super-resolution structural imaging microscope (Evident Corporation, Tokyo, Japan). Image processing and analysis were performed using HIS-SIM Image software (version 1.4.23a).

According to the MISEV2023 guidelines, characterization of apoVs was based on multiple complementary criteria: (1) morphology visualized by TEM, (2) size distribution measured by NTA, (3) phosphatidylserine exposure detected by Annexin V positivity, and (4) isolation via sequential centrifugation at 17,500× *g*, which selectively enriches for large EVs from apoptotic cells. This multi-parameter approach confirms the identity of apoVs as a distinct EV population, not solely defined by particle size [44].

### 4.8. CCK8 Analysis

Cell viability was assessed using the Cell Counting Kit-8 (CCK-8; KeyGen Bio, Nanjing, China) according to the manufacturer’s instructions. MSCs were seeded into 96-well plates at a density of 2000 cells per well. Subsequently, cells were co-cultured with either apoVs or exosomes at concentrations of 1 × 10^6^ or 2 × 10^6^ particles. At the indicated time points (1, 2, and 3 days), the cells were washed once with PBS and incubated in the dark for 2 h in a mixture of 100 μL culture medium and 10 μL CCK-8 solution. Absorbance was measured at 450 nm using a Spark multimode microplate reader (TECAN, Männedorf, Switzerland).

### 4.9. SA-β-Galactosidase Staining

Cellular senescence in mesenchymal stem cells was assessed using a β-galactosidase staining kit (KeyGen Bio, China) according to the manufacturer’s protocol. The percentage of senescent cells was determined by calculating the ratio of positively stained cells to the total number of cells across five randomly selected fields of view.

### 4.10. EdU Proliferation Experiment

Cell proliferation was assessed using the kFluor488-EdU Cell Proliferation Detection Kit (KeyGEN Bio, China). hBMMSCs were seeded into 96-well plates (BIOFIL, China) at a density of 2000 cells per well and co-cultured with 2 × 10^6^ apoVs or exosomes for the designated durations. Subsequently, cells were incubated with 50 μM EdU for 2 h. After fixation with 4% paraformaldehyde (PFA, ServiceBio, China) at room temperature for 15 min, staining was performed according to the manufacturer’s instructions. Images were captured using Ti2-A fluorescence microscope (Nikon, Tokyo, Japan), and the number of proliferating cells was quantified in at least five randomly selected fields per sample.

### 4.11. Osteogenic Differentiation Assay

For osteogenic differentiation, hBMMSCs were seeded at a density of 5 × 10^4^ cells/well in 24-well plates. Upon reaching 95% confluence, the culture medium was switched to osteogenic induction medium consisting of α-MEM supplemented with 15% FBS, 1% P/S, 1.8 mM potassium dihydrogen phosphate (Solarbio, Beijing, China), 100 μM L-ascorbic acid phosphate (Gibco), 2 mM β-glycerophosphate (Gibco), and 10 nM dexamethasone (Yeasen, Shanghai, China). After 14–21 days of induction, cells were fixed with 4% PFA, stained with 1% Alizarin Red S (Yeasen), and analyzed using ImageJ software (version 1.54p). Additionally, for Western blotting analysis, total protein was extracted from cells 10 days after the initiation of osteogenic induction.

### 4.12. Scratch Wound-Healing Assay

Cell migration of hBMMSCs following treatment with apoVs or exosomes was evaluated using a scratch wound-healing assay. hBMMSCs were seeded into 6-well plates at a density sufficient to achieve 100% confluence by the second day. A linear scratch was created across the cell monolayer, perpendicular to the reference grid lines on the bottom of the plate, using a sterile 200-μL pipette tip. Images of the wound area were captured at 0, 12, and 24 h to quantify the extent of cell migration.

### 4.13. Micro CT Analysis

Mice used in the OVX model were 10 weeks old at the time of surgery. Therapeutic intervention was initiated 12 weeks post-surgery. Following our previous protocol [45], mice received weekly intravenous injections for two consecutive months. We administered a protein dosage of 20 µg for both vesicle types or an equivalent volume of PBS. This corresponds to approximately 1.0 × 10^10^ particles of apoVs and 4 × 10^10^ particles of exosomes. The mice were euthanized at 40 weeks of age. Subsequently, femurs were harvested, fixed in 4% paraformaldehyde, and analyzed using a Venus Micro-CT system (PINGSENG Healthcare, Kunshan, China). The scanning parameters were set to a tube voltage of 80 kV and a tube current of 0.1 mA. Data visualization and analysis were performed using Avatar software (PINGSENG Healthcare, Kunshan, China; https://www.pingseng.com/product/default_28.htm, accessed on 10 May 2026).

### 4.14. Histological Analysis

Following decalcification with 10% ethylenediaminetetraacetic acid (EDTA, pH 7.0) for one month, the distal femurs were embedded in paraffin. Sections were prepared, dewaxed, and subjected to hematoxylin and eosin (H&E) staining, Masson’s trichrome staining, and tartrate-resistant acid phosphatase (TRAP) staining. Finally, the stained sections were imaged using a SWe-CX63 microscope (ServiceBio, China).

### 4.15. miRNA Differential Analysis

Total RNA was isolated using the Total RNA Purification Kit (LC Sciences, Houston, TX, USA) following the manufacturer’s instructions. The concentration and purity of the RNA were assessed using an Agilent 2100 Bioanalyzer with the RNA 6000 Nano LabChip Kit (Agilent, Santa Clara, CA, USA). Small RNA libraries were generated using the TruSeq Small RNA Sample Preparation Kit (Illumina, San Diego, CA, USA). Finally, single-end sequencing (1 × 50 bp) was conducted on an Illumina HiSeq 2500 platform at LC-BIO (Hangzhou, China). following the manufacturer’s recommended workflow. Bioinformatic analyses and data visualization were performed using the R statistical computing environment (version 4.5.1) within RStudio.

### 4.16. RNA Extraction and Quantitative Real-Time PCR (RT-qPCR)

Total RNA was extracted from the treated cells using a Total RNA Extraction Kit (Thermo Fisher Scientific, Waltham, MA, USA) in accordance with the manufacturer’s instructions. First-strand cDNA was subsequently synthesized using a First Strand cDNA Synthesis Kit (Thermo Fisher Scientific). To quantify the expression levels of the candidate miRNAs, quantitative real-time PCR was performed on a CFX96™ Real-Time PCR System (Bio-Rad, Hercules, CA, USA). The amplification was carried out using the miScript SYBR Green PCR Kit along with specific miScript Primer Assays (Qiagen, Hilden, Germany), which consist of target-specific primers designed based on the latest miRBase sequences. snRNA U6 was utilized as the sole endogenous control for miRNA normalization. The relative gene expression levels were calculated using the standard 2^−ΔΔCt^ method. All quantitative PCR analyses were performed in independent triplicates.

### 4.17. ELISA

To evaluate the secretion of Senescence-Associated Secretory Phenotype (SASP) factors, the conditioned medium from the treated hBMMSCs was collected and centrifuged at 1000× *g* for 20 min to completely remove cellular debris. The concentrations of pro-inflammatory cytokines in the supernatants were subsequently quantified using specific commercial ELISA kits in strict accordance with the manufacturers’ instructions. Specifically, the levels of Interleukin-6 (IL-6) and Tumor Necrosis Factor-alpha (TNF-α) were measured using the Human IL-6 Uncoated ELISA Kit (Cat. No. 88-7066, Invitrogen, USA) and the Human TNF alpha Uncoated ELISA Kit (Cat. No. 88-7346, Invitrogen, USA), respectively. The concentration of MCP-1 was determined using the Human MCP1 ELISA Kit (Cat. No. SEA087Hu, Cloud-Clone Corp., Katy, TX, USA). The optical density (OD) of each well was immediately measured at a wavelength of 450 nm using a microplate spectrophotometer. The absolute concentrations of the cytokines were calculated based on the respective standard curves generated during each independent assay. All samples were analyzed in independent triplicates.

### 4.18. Western Blot

Total cellular proteins were extracted using RIPA Lysis Buffer (Beyotime, Shanghai, China) supplemented with a protease and phosphatase inhibitor cocktail (CWBIO, Taizhou, China) to strictly preserve protein phosphorylation states. Protein concentrations were quantified using a BCA Protein Assay Kit (Biosharp, Beijing, China). Equal amounts of protein (20 μg per lane, previously optimized to fall within the linear detection range) were resolved by sodium dodecyl sulfate-polyacrylamide gel electrophoresis (SDS-PAGE) using FuturePAGE™ precast gels (ACE Bio, Xiangtan, China) and electro-transferred onto Polyvinylidene Fluoride (PVDF) membranes. To assess phosphorylation, parallel gels were loaded with identical samples and probed separately for phosphorylated and total proteins. The membranes were blocked with Bovine Serum Albumin (BSA, Beyotime) and incubated overnight at 4 °C with the following primary antibodies (all diluted at 1:5000): anti-CRKL (Abcam, Waltham, MA, USA), anti-p16 (Affinity Biosciences, Cincinnati, OH, USA), anti-p21 (Abcam, USA), and anti-BRAF, anti-p38, anti-phospho-p38, anti-JNK, anti-phospho-JNK, anti-p65, and anti-phospho-p65 (Proteintech Group, Shanghai, China). Anti-β-actin (1:5000, Proteintech Group) served as the internal loading control. After washing, the membranes were incubated with HRP-conjugated secondary antibodies. Immunoreactive protein bands were visualized using an ultrasensitive ECL chemiluminescent substrate (Meilunbio, Dalian, China). The optical densities of the bands were quantified using ImageJ software (version 1.54p, NIH, Bethesda, MD, USA). For analytical normalization, the relative levels of phosphorylated proteins were normalized to their respective total protein levels, whereas other target proteins were normalized to β-actin.

### 4.19. Statistical Analysis

Statistical analyses were performed using GraphPad Prism 8.0 software. Data are presented as mean ± standard deviation (SD). For comparisons involving multiple groups, statistical significance was determined using one-way analysis of variance (ANOVA) followed by Tukey’s post hoc test. Clinical parameters were compared using paired Student’s *t*-tests. * *p* < 0.05 was considered statistically significant. Significance levels are denoted as follows: * *p* < 0.05, ** *p* < 0.01, *** *p* < 0.001, and **** *p* < 0.0001. “ns” indicates no significance.

## 5. Conclusions

This study demonstrates that mesenchymal stem cell-derived apoptotic vesicles attenuate postmenopausal osteoporosis more effectively than conventional exosomes. This enhanced efficacy is associated with a reduced senescence-associated secretory phenotype (SASP) in hBMMSCs and is attributed to a specific microRNA cluster (hsa-let-7b-5p, hsa-miR-92a-3p, and hsa-miR-98-5p) enriched in apoVs compared to exosomes. These miRNAs inhibit both MAPK and NF-κB signaling, accompanied by reduced BRAF and CRKL protein expression, thereby reducing age-related inflammation and preserving osteogenic function. Our findings suggest that apoptotic vesicles represent a promising cell-free therapeutic strategy for age-related bone loss, offering strong translational advantages over exosome-based approaches.

## Data Availability

The original contributions presented in this study are included in the article/Supplementary Materials. Further inquiries can be directed to the corresponding authors.

## Supplementary Materials

The following supporting information can be downloaded at: https://www.mdpi.com/article/10.3390/ph19050777/s1, Figure S1: Characterization of hBMMSCs.
