# Supplementary material for: MSC-Derived Apoptotic Vesicles Restore Bone Marrow Niche Homeostasis in Postmenopausal Osteoporosis by miRNA-Mediated Suppression of MAPK and NF-κB Signaling Nodes
Source: Pharmaceuticals (Basel). 2026 May 15;19(5):777. doi: 10.3390/ph19050777 (PMC13210132; doi:10.3390/ph19050777)
Supplement: Supplementary file 1 [file pharmaceuticals-19-00777-s001.zip › pharmaceuticals-4269621-supplementary.pdf]

Supporting Information for

MSC-Derived Apoptotic Vesicles Restore Bone Marrow Niche Homeostasis in Postmenopausal Osteoporosis by miRNA-Mediated Suppression of MAPK and NF-κB Signaling Nodes

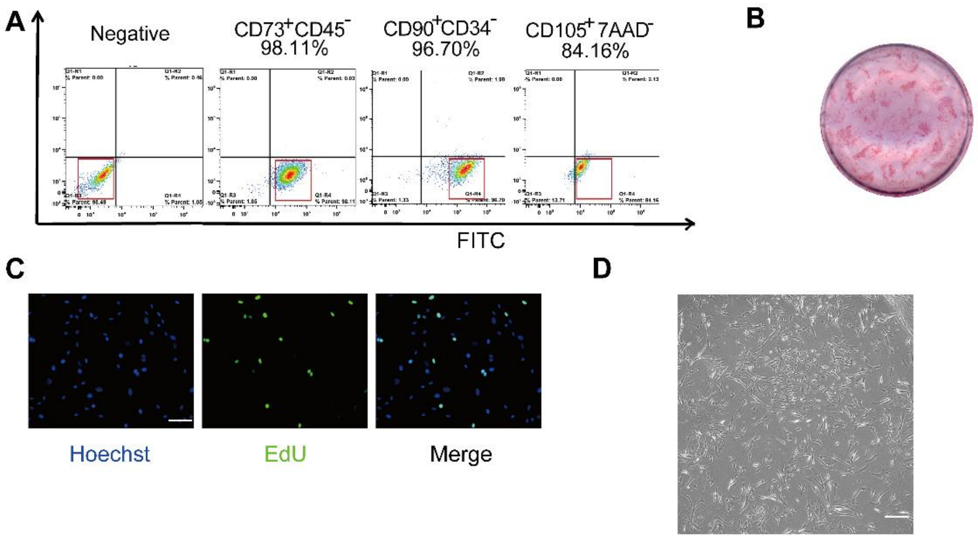

Figure Legends

Figure S1 Characterization of hBMMSCs. (A) Flow cytometry analysis of hBMMSCs showing the expression of key surface markers, including CD34, CD45, CD73, CD90, and CD105; (B) Alizarin Red S staining indicating the osteogenic differentiation potential of hBMMSCs (red deposits indicate calcium accumulation, n=4 per group, sacle bar: 400 μm); (C) Proliferation of hBMMSCs was assessed using the EdU assay, n = 3 per group. Scale bar, 100 μm; (D) The morphology of control hBMMSCs. Scale bar, 100 μm. Each experiment was repeated at least three times.
